# Reduced amygdala reactivity and impaired working memory during dissociation in borderline personality disorder

**DOI:** 10.1007/s00406-017-0806-x

**Published:** 2017-05-19

**Authors:** Annegret Krause-Utz, Dorina Winter, Friederike Schriner, Chui-De Chiu, Stefanie Lis, Philip Spinhoven, Martin Bohus, Christian Schmahl, Bernet M. Elzinga

**Affiliations:** 1 Department of Psychosomatic Medicine and Psychotherapy, Central Institute of Mental Health (CIMH), Mannheim, Germany; 2 Medical Faculty, University of Heidelberg, Mannheim, Germany; 3 Institute of Clinical Psychology, Leiden University, Leiden, The Netherlands; 4 Leiden Institute for Brain and Cognition (LIBC), Leiden, The Netherlands; 5 Department of Psychology, Centre for Cognition and Brain Studies, and Clinical and Health Psychology Centre, The Chinese University of Hong Kong, Shatin, Hong Kong; 6 Institute of Psychiatric and Psychosomatic Psychotherapy, CIMH, Mannheim, Germany; 7 Department of Psychiatry, Leiden University Medical Center, Leiden, The Netherlands

**Keywords:** Borderline personality disorder, Working memory, Memory, Neuroimaging, Stress

## Abstract

Affective hyper-reactivity and impaired cognitive control of emotional material are core features of borderline personality disorder (BPD). A high percentage of individuals with BPD experience stress-related dissociation, including emotional numbing and memory disruptions. So far little is known about how dissociation influences the neural processing of emotional material in the context of a working memory task in BPD. We aimed to investigate whole-brain activity and amygdala functional connectivity (FC) during an Emotional Working Memory Task (EWMT) after dissociation induction in un-medicated BPD patients compared to healthy controls (HC). Using script-driven imagery, dissociation was induced in 17 patients (‘BPD_D’), while 12 patients (‘BPD_N’) and 18 HC were exposed to neutral scripts during fMRI. Afterwards, participants performed the EWMT with neutral vs. negative IAPS pictures vs. no distractors. Main outcome measures were behavioral performance (reaction times, errors) and whole-brain activity during the EWMT. Psychophysiological interaction analysis was used to examine amygdala connectivity during emotional distraction. BPD patients after dissociation induction showed overall WM impairments, a deactivation in bilateral amygdala, and lower activity in left cuneus, lingual gyrus, and posterior cingulate than BPD_N, along with stronger left inferior frontal gyrus activity than HC. Furthermore, reduced amygdala FC with fusiform gyrus and stronger amygdala FC with right middle/superior temporal gyrus and left inferior parietal lobule was observed in BPD_D. Findings suggest that dissociation affects reactivity to emotionally salient material and WM. Altered activity in areas associated with emotion processing, memory, and self-referential processes may contribute to dissociative states in BPD.

**Electronic supplementary material:**

The online version of this article (doi:10.1007/s00406-017-0806-x) contains supplementary material, which is available to authorized users.

## Introduction

Borderline personality disorder (BPD) is a severe mental disorder, characterized by emotion dysregulation, instable cognitions, impulsivity, interpersonal disturbances, and dissociation [1–6]. Previous neuroimaging studies in BPD suggest that a hyper-reactivity and hyper-connectivity of the amygdala may underlie disturbed emotion processing in BPD [7, 8], although discrepant findings were also reported [9]. The amygdala plays a crucial role in the initiation of fear and stress responses [10] and might also be involved in stress-related dissociation [11].

Dissociation occurs in a high percentage (~75–80) of individuals with BPD, involving disruptions in the usually integrated functions of consciousness, perception, identity, memory, and affect and has been closely linked to psychological trauma [6, 12–17]. Dissociative symptoms such as depersonalization, derealization, numbing, and analgesia may provide a state of subjective detachment from extremely stressful experiences, e.g., by dampening overwhelming emotions and reducing awareness of pain [15, 17]. In pathological dissociation, the cost of this subjective detachment is a disruption of executive functions that are crucial to goal-directed behavior, such as attention, learning, and memory. More specifically, dissociation may hinder the conscious processing and integration of salient information in autobiographical memory, which can have detrimental effects on the development of identity and emotion regulation capacities. Dissociation may hinder the recall and learning of self-relevant information also during therapy [13, 24] and in BPD, dissociative symptoms predicted poor treatment outcome [18, 19]. However, the precise neuropsychological mechanisms underlying this relationship remain unclear.

Neurobiological models have linked dissociation to a dampened activity in the amygdala and increased recruitment of ‘cognitive control’ regions, such as the medial prefrontal cortex (mPFC), anterior cingulate cortex (ACC), and inferior frontal gyrus [15, 20] as well as to altered activity in the superior temporal gyrus, precuneus, posterior cingulate, which are implicated in autobiographical memory and self-referential processing [21–23]. The amygdala appears to be an important hub within this network, sharing strong functional connections with the ACC, insular and orbitofrontal cortex, mPFC, parahippocampal gyrus, precuneus, posterior cingulate, among others [24, 25]. In summary, it can be assumed that dissociation substantially affects activity within an ‘amygdala network’ involved in the processing of self-relevant emotional information and the initiation of stress responses. In BPD, however, so far there is little empirical evidence for this.

Only few neuroimaging studies in BPD so far investigated associations between self-reported dissociation and brain activity during experimental challenge, such as the presentation of aversive images or negative words [26–29]. To the best of our knowledge, only two neuroimaging studies in BPD used script-driven imagery to more directly investigate the effect of experimentally induced dissociation on brain activity [21, 29]. In this well-established paradigm, a narrative of an autobiographical situation involving dissociative experiences (‘dissociation script’) is created and presented in an experimental setting, e.g., during functional magnetic resonance imaging (fMRI). Participants are instructed to listen to this script and to recall their autobiographical experiences as vividly as possible [23], which successfully induced dissociation in previous research [21, 29]. When exposed to a dissociation script compared to a neutral script, BPD patients showed significantly increased activity in the left inferior frontal gyrus and diminished temporo-limbic activity, which was even more pronounced in a subgroup of traumatized patients [21]. We recently combined script-driven imagery with an Emotional Stroop Task (EST), to investigate the effect of a dissociation induction on interference inhibition, on a behavioral and neural level [29]. BPD patients exposed to a dissociation script showed impaired accuracy and slower reaction times for negative words than patients exposed to a neutral script. Patients after dissociation induction further showed increased left superior frontal activity in response to negative vs. neutral words [29]. However, it remains unclear how brain areas may interact during affective–cognitive processing after dissociation induction in BPD.

Moreover, to our knowledge, no study in BPD so far investigated how dissociation affects the neural processing of emotional material in the context of a working memory task, which requires the conscious manipulation of task-irrelevant stressful information. We previously used a modified version of the Emotional Working Memory Task (EWMT) in which task-irrelevant neutral vs. negative interpersonal pictures from the International Affective Picture System (IAPS) [30] or only a fixation cross (i.e., no distractors) are presented during the delay interval of a Sternberg item recognition task [27, 28]. Participants are instructed to ignore distractors, focusing solely on the WM task, thereby voluntarily inhibiting emotion processing in favor of cognitive processing. WM impairments and amygdala reactivity to negative pictures were significantly stronger in BPD patients, suggesting increased emotional distractibility compared to healthy controls (HC) [27]. During emotional distraction, BPD patients further showed a stronger coupling of the amygdala with the hippocampus and dorsomedial PFC, suggesting enhanced self-referential processing [28].

Here, we aimed to investigate the impact of experimentally induced dissociation on brain activity and amygdala functional connectivity during the EWMT. Studying this relationship on a behavioral and neural level might help to shed more light on the effects of stress-related dissociation in BPD. Script-driven imagery was used to induce dissociation. For patients exposed to a neutral script, we hypothesized to replicate previous findings of amygdala hyper-reactivity to emotional pictures, while patients exposed to a dissociation script were expected to show significantly dampened amygdala reactivity and increased activity in frontal areas (inferior frontal gyrus, medial prefrontal cortex, anterior cingulate cortex).

## Materials and methods

### Sample

Sixty women aged between 18 and 45 years (40 patients with BPD according to DSM-IV [16] and 20 female HC) participated. BPD patients were recruited via advertisement on websites or referred from the residential treatment unit of the Department of Psychosomatic Medicine and Psychotherapy at the Central Institute of Mental Health (CIMH) in Mannheim, Germany. HC were recruited via newspaper advertisements. General exclusion criteria were serious somatic illnesses, traumatic brain injuries, developmental disorders, and MRI-related criteria (metal implants, pregnancy, left-handedness, claustrophobia). Exclusion criteria for HC were lifetime history of Axis-I/II disorders. Specific exclusion criteria for patients were psychotropic medication within 4 weeks prior to the study, substance dependence during the last year, substance abuse within 2 months prior to participation, current/lifetime psychotic or bipolar-I disorder, and life-threatening suicidal crisis.

Patients were randomly assigned to two experimental conditions: 20 patients were exposed to a dissociation script (‘BPD_D’), while 20 BPD patients (‘BPD_N’) and 20 HC were exposed to a neutral script. An increase of ≥1.5 scores on the Dissociation Stress Scale 4 (DSS-4, see below) [31] after script compared to baseline was defined as inclusion criterion for the BPD_D group (criterion was met by all participants assigned to this group). To ensure that individuals in the BPD_N group were not highly dissociated, we excluded patients with DSS-4 scores of ≥3 at baseline and/or an increase of >1.5 scores after the experiment (three patients had to be excluded for this reason). Part of the collected data had to be discarded due to movement artifacts during fMRI (BPD_N: *n* = 2, BPD_D: *n* = 3, HC: *n* = 2), technical problems during script presentation (BPD_N: *n* = 1), or inconsistent button presses (95–100% errors, indicated that task instructions were not understood correctly in 2 BPD_N). The final sample comprised 17 BPD_D, 12 BPD_N, and18 HC.

Clinical diagnoses were assessed by trained diagnosticians using the Structured Clinical Interview for DSM-IV Axis-I Disorders (SCID-I) [32] and International Personality Disorder Examination (IPDE) [33]. Further clinical assessment included questionnaires on symptom severity (Borderline Symptom List 23, BSL-23 [34]), childhood abuse/neglect (Childhood Trauma Questionnaire, CTQ [35]), trait dissociation (Dissociative Experiences Scale, DES [36]), depressive symptoms (Beck Depression Inventory II, BDI-II [37]), state anxiety (State Anxiety Questionnaire, STAI [38]), and Attention-Deficit Hyperactivity Disorder symptoms (childhood: Wender Utah Rating Scale, WURS [39], adulthood: ADHD-Checklist [40]).

The groups did not differ significantly regarding age and years of education (Table 1A). Both BPD groups scored significantly higher than HC on clinical measures but did not differ significantly from each other; all patients reported at least one type of severe to extreme childhood abuse and/or neglect. Criteria for comorbid Posttraumatic Stress Disorder (PTSD) were met by 7 BPD_D patients (41%) and 5 BPD_N patients (41%), i.e., were distributed equally in both BPD groups. Further comorbidities and clinical characteristics of the two BPD groups are presented and compared in Table 1B. Dissociative states were induced using script-driven imagery and measured by the DSS-4, a self-rating scale with excellent internal consistency and reliability, high specificity, and sensitivity to change in symptomatology [31]. The DSS-4 consists of four items on current psychological (derealisation, depersonalization) and somatic (pain perception, hearing) dissociation and one item on current aversive tension (10-point Likert scales, 0 = not at all, 9 = extremely).

**Table 1:** Demographic variables, dissociation and arousal ratings, and clinical characteristics

(A)	BPD_D	BPD_N	HC	
Age (years)	27.41 ± 6.20	25.17 ± 6.21	29.61 ± 8.61	*F*_(2,44)_ = 1.38, *p* = 0.262
Years of education	10.59 ± 2.62	10.08 ± 3.03	10.72 ± 1.99	*F*_(2,44)_ = 0.25, *p* = 0.784
DSS-4				
Dissociation ratings baseline	3.44 ± 1.99	2.30 ± 1.14	1.31 ± 0.66	*F*_(2,42)_ = 11.27, *p* < 0.0001. BPD_D-HC: 2.26, *p* < 0.0001 BPD_N-HC: 1.00, *p* = 0.160 BPD_D-BPD_N: 1.27, *p* = 0.062
Dissociation ratings after script	6.85 ± 2.03	1.85 ± 0.84	1.19 ± 0.51	*F*_(2,42)_ = 92.50, *p* < 0.0001 BPD_D-HC: 5.79, *p* < 0.0001 BPD_N-HC: 0.60, *p* = 0.465 BPD_D-BPD_N: 5.19, *p* < 0.0001
Arousal rating baseline	4.76 ± 2.36	3.91 ± 1.97	2.72 ± 2.02	*F*_(2,42)_ = 3.43, *p* = 0.042 BPD_D-HC: 1.90, *p* = 0.035 BPD_N-HC: 1.20, *p* = 0.325 BPD_D-BPD_N: 0.72, *p* = 0.672
Arousal rating after script	7.71 ± 2.11	4.50 ± 2.65	2.17 ± 2.28	*F*_(2,42)_ = 26.67 *p* < 0.0001 BPD_D-HC: 5.46, *p* < 0.0001 BPD_N-HC: 1.83, *p* = 0.840 BPD_D-BPD_N: 3.62, *p* < 0.0001
BSL-23 total score (BPD symptom severity)	47.12 ± 19.23	43.33 ± 13.36	1.33 ± 1.81	*F*_(2,44)_ = 60.51, *p* < 0.0001, ƒ^2^ = 0.73 BPD_D-HC: 45.78, *p* < 0.0001 BPD_N-HC: 42.00, *p* < 0.0001 BPD_D-BPD_N: 3.78, *p* = 0.737
DES total score (trait dissociation)	31.74 ± 16.52	26.93 ± 13.50	2.68 ± 2.04	*F*_(2,44)_ = 28.37, *p* < 0.0001, ƒ^2^ = 0.56 BPD_D-HC: 29.01, *p* < 0.0001 BPD_N-HC: 24.26, *p* < 0.0001 BPD_D-BPD_N: 4.81, *p* = 0.547
BDI-II (depressive symptoms)	24.47 ± 11.89	26.75 ± 10.68	1.67 ± 2.25	*F*_(2,44)_ = 38.49, *p* < 0.0001, ƒ^2^ = 0.64 BPD_D-HC: 22.80, *p* < 0.0001 BPD_NHC: 25.08, *p* < 0.0001 BPD_D-BPD_N: 2.28, *p* = 0.783
STAI state^a^ (state anxiety)	56.19 ± 10.13	52.92 ± 6.36	29.39 ± 5.41	*F*_(2,43)_ = 54.90, *p* < 0.0001, ƒ^2^ = 0.74 BPD_D-HC: 26.79, *p* < 0.0001 BPD_ N-HC: 23.53, *p* < 0.0001 BPD_D-BPD_N: 2.55, *p* = 0.503
STAI trait^a^ (trait anxiety)	58.13 ± 7.03	60.58 ± 5.83	28.72 ± 4.66	*F*_(2,43)_ = 138,83, *p* < 0.0001, ƒ^2^ = 0.87 BPD_D-HC: 29.40, *p* < 0.0001 BPD_N-HC: 31.86, *p* < 0.0001 BPD_D-BPD_N: 2.05, *p* = 0.522
WURS (childhood ADHD symptoms)	98.80 ± 41.16	94.42 ± 27.91	49.53 ± 27.52	*F*_(2,39)_ = 9.88, *p* < 0.0001, ƒ^2^ = 0.39 BPD_D-HC: 49.27, *p* < 0.0001 BPD_N -HC: 44.88, *p* < 0.0001 BPD_D-BPD_N: 4.39, *p* = 0.938
ADHD checklist^a^ (adult ADHD symptoms)	14.94 ± 9.80	16.83 ± 8.33	3.94 ± 2.88	*F*_(2,44)_ = 14.11, *p* < 0.0001, ƒ^2^ = 0.39 BPD_D-HC: 10.99, *p* < 0.0001 BPD_N-HC: 12.89, *p* < 0.0001 BPD_D-BPD_N: 1.89, *p* = 0.789
CTQ total sum-score (childhood abuse and neglect)	68.23 ± 25.12	70.58 ± 16.46	33.39 ± 11.88	*F*_(2,44)_ = 20.34, *p* < 0.0001, ƒ^2^ = 0.48 BPD_D-HC: 34.91, *p* < 0.0001 BPD_N-HC: 37.19, *p* < 0.0001 BPD_D-BPD_N: 2.29, *p* = 0.944

(B) Clinical characteristics and comorbidities *n* (%)		BPD_D (*n* = 17)	BPD_N (*n* = 12)	*χ* ^2^ tests
BPD criteria fulfilled	(DSM-IV)			
Fear of abandonment	1	9 (53%)	12 (100%)	*χ* ^*2*^ = 0.37, *p* = 0.830
Instable relationships	2	8 (47%)	8 (67%)	*χ* ^*2*^ = 1.60, *p* = 0.450
Identity disturbance	3	10 (59%)	10 (83%)	*χ* ^*2*^ = 3.53, *p* = 0.171
Impulsivity	4	7 (41%)	5 (42%)	*χ* ^*2*^ = 0.52, *p* = 0.773
Non-suicidal self-injury	5	14 (82%)	8 (67%)	*χ* ^*2*^ = 1.08, *p* = 0.583
Affective instability	6	17 (100%)	12 (100%)	–
Emptiness	7	12 (71%)	9 (75%)	*χ* ^*2*^ = 0.73, *p* = 0.695
Anger	8	17 (100%)	8 (67%)	*χ* ^*2*^ = 2.31, *p* = 0.316
Dissociation	9	17 (100%)	12 (100%)	–
Self-injurious behavior (last 12 month)		15 (88%)	10 (83%)	*χ* ^*2*^ = 0.14, *p* = 0.706
Major depressive disorder	Current	2 (12%)	0 (0%)	*χ* ^*2*^ = 1.49 *p* = 0.223
	Lifetime	15 (88%)	8 (66%)	*χ* ^*2*^ = 2.28, *p* = 0.131
Dysthymia	Current	0 (0%)	1 (8%)	*χ* ^*2*^ = 1.51, *p* = 0.219
Panic disorder	Current	3 (18%)	2 (17%)	*χ* ^*2*^ = 0.01, *p* = 0.970
	Lifetime	5 (29%)	3 (18%)	*χ* ^*2*^ = 0.05, *p* = 0.824
Social phobia	Current	8 (47%)	2 (17%)	*χ* ^*2*^ = 2.83, *p* = 0.093
	Lifetime	10 (59%)	4 (33%)	*χ* ^*2*^ = 1/78, *p* = 0.182
Specific phobia	Current	3 (18%)	1 (8%)	*χ* ^*2*^ = 0.48, *p* = 0.488
	Lifetime	3 (18%)	1 (8%)	*χ* ^*2*^ = 0.48, *p* = 0.488
Obsessive compulsive disorder	Lifetime	4 (24%)	1 (8%)	*χ* ^*2*^ = 1.09, *p* = 0.296
Posttraumatic stress disorder	Current	7 (41%)	5 (41%)	–
	Lifetime	8 (47%)	5 (41%)	*χ* ^*2*^ = 0.88, *p* = 0.646
Somatization disorder	Lifetime	1 (6%)	0 (0%)	*χ* ^*2*^ = 0.71, *p* = 0.398
Eating disorders	Current	1 (6%)	1 (8%)	–
	Lifetime	7 (41%)	3 (18%)	*χ* ^*2*^ = 0.76, *p* = 0.384
Drug abuse	Lifetime	2 (12%)	1 (8%)	*χ* ^*2*^ = 0.15, *p* = 0.929
Alcohol abuse	Lifetime	1 (6%)	0 (0%)	*χ* ^*2*^ = 1.52, *p* = 0.468
Previous medication		13 (76%)	9 (75%)	*χ* ^*2*^ = 0.008, *p* = 0.927
Acamprosate		0 (0%)	1 (8%)	
Atypical antipsychotics		1 (6%)	1 (8%)	
BZD		2 (12%)	1 (8%)	*χ* ^*2*^ = 6.21, *p* = 0.400
SNRI		3 (18%)	2 (17%)	
SSRI		6 (35%)	1 (8%)	
TCA		1 (6%)	3 (18%)	
Time of last medication^b^				
1 month ago		3 (18%)	1 (8%)	
≥3 month ago		2 (12%)	1 (8%)	
≥6 month ago		2 (12%)	6 (50%)	*χ* ^*2*^ = 4.76, *p* = 0.190
≥12 month ago		4 (24%)	1 (8%)	

*M* mean, *SD* standard deviation, *DSS-4* Dissociation Stress Scale 4, *BPD_D* patients with borderline personality disorder exposed to a dissociation script, *BPD_N* patients with borderline personality disorder exposed to a neutral script, *HC* healthy controls, *BSL-23* borderline, *BZD* benzodiazepine, *SSRI* selective serotonin reuptake inhibitor, *SNRI* serotonin–norepinephrine reuptake inhibitor, *TCA* Tricyclic antidepressant. Symptom List 23, *DES* Dissociative Experience Scale, *BDI* Beck Depression Inventory, *STAI* State Anxiety Inventory, *CTQ* Childhood Trauma Questionnaire, *WURS* Wender Utah Rating Scale

^a^STAI scores in one BPD_D patient and WURS scores in 3 HC and 2 BPD_D patients were missing

^b^Information in 2 BPD_D patients was missing

### Emotional Working Memory Task (EWMT)

The EWMT was a validated Sternberg item recognition task [41], modified by Oei and colleagues [42, 43]. Our adapted version [27] consisted of 48 trials, each starting with a set of three uppercase letters (memoranda, 1000 ms), followed by a delay interval (1500 ms), and a probe (thee uppercase letters, 2000 ms). In half of the trials, one of the three memoranda was present in the probe. Participants had to press a ‘yes’ or ‘no’ button indicating whether they had recognized a target or not. During the delay interval either no distractors (only a fixation cross) or neutral vs. negative distractors (interpersonal scenes from the IAPS, selected based on arousal and valance ratings in the general population [30]) were presented. Negative pictures depicted scenes of interpersonal violence (e.g., sexual attack, physical assault, beaten/frightened child, physically mutilated body). Neutral pictures included interpersonal scenes with similar complexity (e.g., people at a market place or supermarket). Trials without distractors (only a fixation cross) were added, as even neutral interpersonal stimuli were found to be perceived as emotionally arousing in individuals with BPD, increasing amygdala activity [27]. In addition, participants performed 15 trials of the basic Sternberg paradigm without distractors (i.e., only a fixation cross) to assess baseline working memory. Target-present and target-absent trials were equal in all conditions and balanced in a pseudo-random manner. Software Presentation (Neurobehavioural systems, http://www.neurobs.com/) was used to present stimuli and record behavioral data.

### Procedure

The experiment was approved by the local ethics committee (Medical Faculty of Heidelberg University) and conducted at the CIMH in Mannheim, Germany. All participants received information about the study and scanning procedure, signed written informed consent, and underwent diagnostic and clinical assessment. Then, participants prepared a personalized script of 30-s length together with one experimenter (F.S. and D.W.). Patients assigned to the BPD_D group were instructed to report a non-trauma-related autobiographical situation involving dissociation. BPD_N and HC were instructed to report an emotionally neutral everyday situation. A person unknown to participants read each script aloud recording it on audio tape. During the experiment, participants first practiced five trials of the EWMT outside the scanner. Inside the scanner, scripts were presented via headphones. DSS-4 ratings were assessed before and after scripts. Then participants performed the EWMT (first the 15 trials of the basic Sternberg paradigm, then the EWMT with and without distractors). Participants were instructed to focus on the middle of the screen, to concentrate on the task only and to ignore distractors. Event-related fMRI data were acquired during ratings, script, and EWMT.

### FMRI scan protocol

MRI was conducted using a 3-Tesla Siemens TRIO-Scanner (Siemens, Erlangen). Head cushions and headphones were used to reduce head movement artifacts and scanning noise. Blood oxygen level-dependent (BOLD) signal was measured with 36 3-mm transversal slices covering the entire brain using gradient echo-planar-imaging (EPI) [T2-weighted contrast, field of view = 192 × 192 mm, voxel size = 3 × 3 × 3 mm^3^, voxel matrix = 64 × 64, flip angle = 80°, spin-echo time = 30 ms, inter-scan repetition time (TR) = 2000 ms]. After fMRI, as individual template for functional data, a high-resolution anatomical scan was acquired using three-dimensional magnetization-prepared rapid acquisition gradient echo (MPRAGE) [T1-weighted contrast, voxel size = 1 × 1 × 1 mm^3^].

### Statistical analysis

Custom statistical software (*SPSS*, Chicago: *SPSS* Inc) was used for manipulation check, behavioral data analysis, and follow-up (subgroup) comparisons. Normal distribution was checked for all variables using the Kolmogorov–Smirnov test. For repeated measurement analysis of variance (rmANOVA), assumptions of variance equality (Levene’s tests) and sphericity (Mauchly’s test) were checked (in case of violations Greenhouse–Geisser corrections were applied). Significant effects were followed up using between-group or paired *t* tests (*p* < 0.05, two tailed).

#### Manipulation check

A 3 × 2 rmANOVA with DSS4-scores before and after script as dependent variables (within-subject factor Time) and Group as between-subject factor was performed to check whether self-reported dissociation significantly changed after script.

#### Behavioral (WM) data

WM data were checked for outliers. Errors were scored as incorrect, too early responses, and misses (omissions) separately. Percentage of incorrect responses as well as reaction times (RTs) for correct trials were analyzed using two separate 3 × 3 rmANOVAs with Group as between-subjects factor and Condition (no distraction vs. neutral vs. negative distractors) as within-subject factor. Differences in specific error types (wrong responses, too early responses, misses) were evaluated using a multivariate ANOVA (MANOVA) with Group as fixed factor. Basic working memory performance (errors, RTs) of trials without distraction was compared between groups using two separate ANOVAs.

#### Fmri data

Functional imaging data were analyzed using standard procedures implemented in the Statistical Parametric Mapping package (SPM8, Neurobehavioral systems, Berkeley, CA; http://www.fil.ion.ucl.ac.uk/spm/). EPI time series were preprocessed according to common standards, including slice time correction, spatial realignment, and unwarping to correct for head motion, co-registration onto participants’ high-resolution T1 scan, normalization to the standard brain of the Montreal Neurological Institute (MNI) space, and smoothing using a Gaussian kernel with a full width at half maximum (FWHM) of 9 mm. Statistical analyses of our event-related design relied upon the general linear model (GLM) to estimate effects of interest [44].

#### Region of interest (ROI) and whole-brain (WB) analysis

*Single subject level* For each participant, task-related activity was identified by convolving a vector of the onset times of the following seven experimental events of interest with a canonical hemodynamic response: memoranda, delay intervals (no, neutral, negative distractors), and probes after no, neutral, and negative distractors, respectively. The GLM further included nuisance variables to control for movement artifacts.

*Group level* To test our a priori hypothesis of decreased amygdala activity in BPD_D, a ROI analysis was conducted using an anatomical mask of the bilateral amygdala (created by the Automated Anatomical Labeling software, AAL [45]), smoothed with a cube of voxels of size (FWHM) of 9 mm. Values of percent signal change in this region during delay intervals (no vs. neutral vs. negative distractors) were extracted for each participant using the rfxplot toolbox [46] and exported to SPSS. Equivalent to the analysis of behavioral data, a 3 × 3 rmANOVA (between-subject factor: Group, within-subject factor: Condition) was then performed in SPSS. To ensure that group differences were not confounded by basic differences in arousal or WM, we repeated the analysis with arousal ratings as well as WM errors as covariate, using two separate rmANCOVAs.

*WB analysis* Consistent with our previous studies [27, 28], a full factorial design was used to model effects of group and experimental task. Within this model, we tested for overall group differences (F contrast) during negative distractors relative to no distractors (as a more neutral control condition [47–49]). Gaussianized *F*/*T* statistic images were determined using a significance threshold of *p* < 0.05, Family-wise error (FWE) corrected for multiple comparisons on the voxel-wise WB level. Based on our a priori hypotheses, small volume corrections (SVC) with pre-defined anatomical masks of the inferior frontal gyrus, mPFC, and ACC (regions of interest) were applied. To follow-up significant WB group effects in subgroup comparisons, parameter estimates were exported to SPSS, and analyzed using between-group *t* tests (*p* < 0.05).

#### Psychophysiological interaction analysis (PPI) analysis

The generalized PPI (gPPI) toolbox by McLaren [50] was applied to analyze changes in the correlation of time series of the amygdala (seed region) with time series of regions across the whole brain, dependent on our experimental manipulation [51, 52]. For the amygdala seed, the same anatomical mask of bilateral amygdala and the same contrast (negative vs. no distractors) as in the above-mentioned ROI analyses were used. For each participant, mean time series of activity from voxels falling within this anatomical mask were extracted and first-level contrasts for the EWMT conditions were computed. Since PPI analysis of event-related designs lacks power [52], increasing the probability of false-negative results (Type-II-error), we decided to apply a more lenient initial clustering threshold of *p* < 0.001, uncorrected on the voxel-wise level (cluster size *k* > 10, *Z* > 3.5). However, only clusters FWE corrected for multiple comparisons (*p* < 0.05) at the cluster level are discussed. PPI beta estimates of significant clusters for negative vs. no distractors (F contrast) were extracted and exported to SPSS. Overall group differences were then evaluated with a MANOVA and followed up using post hoc *t* tests. To ensure that group differences were not confounded by basic differences in WM, we repeated the analysis with WM errors as covariate (MANCOVA).

## Results

### Dissociation induction

Means with standard deviation of DSS-4 scores are reported in Table 1A. Main effects of Time (*F*_(1,43_) = 23.01, *p* < 0.0001, *η* ^*2*^ = 0.35) and Group (*F*_(2,43)_ = 48.57, *p* < 0.0001, *η* ^*2*^ = 0.69) and the interaction effect (*F*_(2,43)_ = 43.79, *p* < 0.0001, *η* ^*2*^ = 0.67) were significant with higher scores after script than baseline in BPD_D (*t*_(16)_ = 7.57, *p* < 0.0001) but not in the other groups (*p* > 0.05).

### Behavioral data

**Fig. 1:**
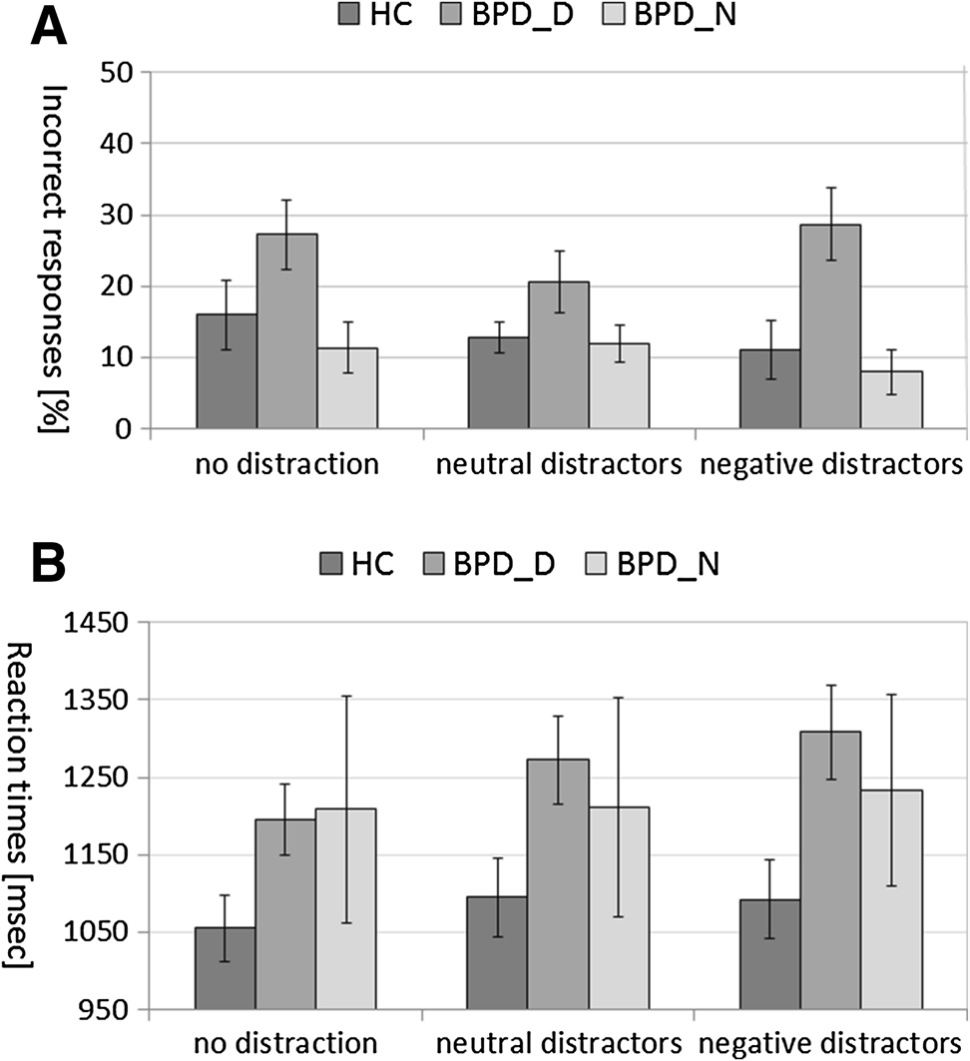
Working memory performance during the Emotional Working Memory Task (after no distraction, after neutral distractors, after negative distractors) in patients with borderline personality disorder (BPD) after dissociation induction (BPD_D) and after the neutral script (BPD_N) as well as in healthy controls (HC). **a** Means ± standard errors of the mean of percentage of errors. **b** Means ± standard errors of the mean of reaction times in correct trials

There were no significant group differences in basic WM (without distractors, *p* > 0.05, data not shown). Figure 1 shows means ± standard errors of the mean (SEM) for percentage of incorrect responses (Fig. 1a) and RTs of correct trials (Fig. 1b) during the EWMT in BPD_D, BPD_N, and HC.

#### Errors during the EWMT

The rmANOVA revealed a significant Group effect (*F*_(2,43)_ = 4.43, *p* = 0.018, *η* ^*2*^ = 0.17) with an overall higher percentage of incorrect responses in BPD_D than in BPD_N (*p* = 0.012) and in HC (*p* = 0.019) (see Fig. 1a). The MANOVA further indicated that there were significant group differences in the number of misses (*F*_(2,43)_ = 6.86, *p* = 0.003, *η* ^*2*^ = 0.24), due to more misses in BPD_D than in BPD_N (*p* = 0.001) and HC (*p* = 0.011), as shown in Supplemental Figure S1.

#### Reaction times during the EWMT

The rmANOVA revealed a significant Condition effect (*F*_(2,42)_ = 4.17, *p* = 0.022, *η* ^*2*^ = 0.17) with longer RTs during neutral (*p* = 0.019) and negative distractors (*p* = 0.003) than during no distractors, but no significant Group effect or interaction effect (both *p* > 0.05) (see Fig. 1b).

### FMRI data

#### ROI analysis

**Fig. 2:**
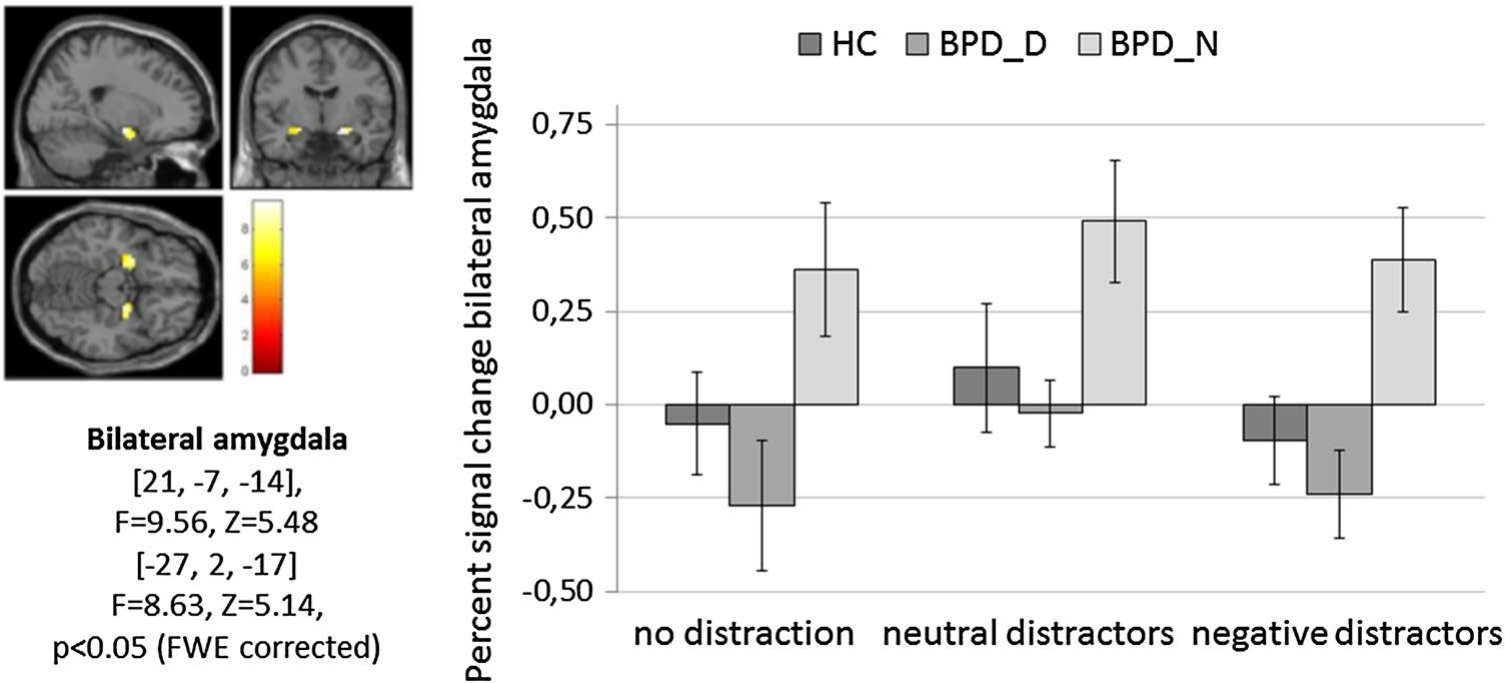
Percent signal change in the bilateral amygdala (region of interest analysis) during the Emotional Working Memory Task (no distraction, neutral distractors, negative distractors) in patients with borderline personality disorder (BPD) after dissociation induction (BPD_D) and after the neutral script (BPD_N) as well as in healthy controls (HC). Clusters in the bilateral amygdala, detected by the main effect of task (*p* < 0.05, FWE corrected on the voxel-wise level) are depicted on the *left*

Figure 2 depicts mean ± SEM of percent signal change in the bilateral amygdala. The rmANOVA revealed a significant main effect for Group (*F*_(2,44)_ = 5.36, *p* = 0.008, ƒ^2^ = 0.20) with higher amygdala activity in BPD_N than in BPD_D (*p* = 0.002) and in HC (*p* = 0.023) (no significant differences between BPD_D and HC, *p* > 0.05). Furthermore, there was a trend for a main effect of Condition (*F*_(2,87)_ = 3.21, *p* = 0.050, ƒ^2^ = 0.13) (interaction effect: *p* > 0.05).

When including self-reported aversive tension (DSS-4 item) as covariate, group differences remained significant (*F*_(2,44)_ = 4.89, *p* = 0.012, ƒ^2^ = 0.19). Likewise, the rmANCOVA with WM errors as covariate still revealed a significant Group effect (*F*_(2,42)_ = 3.43, *p* = 0.042, ƒ^2^ = 0.14) with higher amygdala activity in BPD_N than in BPD_D (*p* = 0.015) and HC (*p* = 0.043).

#### Whole-brain analysis

As a main effect of task (F contrast), there were significant changes in brain activity in the bilateral amygdala, hippocampus, insula, cingulate gyrus, dorsomedial, dorsolateral, ventrolateral prefrontal, occipital, parietal, temporal, and subcortical regions (see Table 2). Significant group differences for brain activity during negative vs. no distractors were found for a cluster comprising left cuneus, lingual gyrus, and posterior cingulate (whole-brain, FWE-corrected *p* < 0.05) and in the left inferior frontal gyrus (BA44) and insula (BA13) (after SVC with the IFC mask). Activity in both clusters was significantly stronger in BPD_N than in HC. Activity in left cuneus, lingual gyrus, and posterior cingulate was also significantly stronger in BPD_N than in BPD_D. In BPD_D, there was significantly stronger activity in left inferior frontal gyrus than in HC (Table 2).

**Table 2:** Results of the full factorial model of brain activity during the Emotional Working Memory Task

F Contrast	Brain region: label (Brodmann area)	Lobe		Cluster size	Peak voxel coordinates (MNI: *X, Y, Z*)	*F* value	*Z* value	*p* value
Main effect of condition	Fusiform gyrus	Occipital Lobe	N.A.	6225	30 −58 −14	31.67	Inf	*p*(FWE) < 0.001
	Fusiform gyrus	Temporal Lobe	BA 20	6225	36 −43 −20	29.10	Inf	*p*(FWE) < 0.001
	Fusiform gyrus	Temporal Lobe	BA 37	6225	42 −49 −17	28.92	Inf	*p*(FWE) < 0.001
	Postcentral gyrus	Parietal Lobe	BA 3	246	−39 −22 52	16.36	7.45	*p*(FWE) < 0.001
	Middle frontal gyrus	Frontal Lobe	BA 6	246	−24 −4 52	12.14	6.31	*p*(FWE) < 0.001
	Cingulate gyrus	Limbic Lobe	BA 32	390	−6 11 46	16.11	7.39	*p*(FWE) < 0.001
	Medial Frontal Gyrus	Frontal Lobe	BA 6	390	−6 −4 55	12.61	6.45	*p*(FWE) < 0.001
	Middle Frontal Gyrus	Frontal Lobe	BA 32	390	9 11 49	12.14	6.32	*p*(FWE) < 0.001
	Insula	Sub-lobar	BA 13	99	−30 23 4	14.92	7.09	*p*(FWE) < 0.001
	Inferior Frontal Gyrus	Frontal Lobe	BA9	173	−54 8 31	13.44	6.69	*p*(FWE) < 0.001
	Inferior Frontal Gyrus	Frontal Lobe	BA9	173	−45 5 31	12.51	6.42	*p*(FWE) < 0.001
	Middle Frontal Gyrus	Frontal Lobe	BA46	173	−48 23 25	7.48	4.68	*p*(FWE) = 0.029
	Insula	Sub-lobar	BA 13	110	36 20 7	12.22	6.34	*p*(FWE) < 0.001
	Dorsolateral prefrontal cortex	?	BA 9	104	45 5 31	12.18	6.33	*p*(FWE) < 0.001
	Putamen	Sub-lobar	Putamen	68	−18 8 −2	12.04	6.29	*p*(FWE) < 0.001
	Amygdala	Limbic Lobe	Amygdala	68	−27 2 −17	8.63	5.14	*p*(FWE) = 0.004
	Middle Frontal Gyrus	Frontal Lobe	BA 6	58	30 −4 52	11.06	5.98	*p*(FWE) < 0.001
	Inferior Parietal Lobule	Parietal Lobe	BA 40	91	−48 −64 40	11.05	5.98	*p*(FWE) < 0.001
	Putamen	Sub-lobar	Putamen	40	21 8 4	10.83	5.91	*p*(FWE) < 0.001
	Inferior Frontal Gyrus	Frontal Lobe	BA 47	85	−42 26 −14	10.57	5.82	*p*(FWE) < 0.001
	Amygdala	Limbic Lobe	Amygdala	65	21 −7 −14	9.56	5.48	*p*(FWE) = 0.001
	Hippocampus	Sub-lobar	Hippocampus	65	30 −10 −17	9.02	5.28	*p*(FWE) = 0.002
	Superior temporal gyrus	Temporal Lobe	BA 22	8	63 −4 4	8.27	5.00	*p*(FWE) = 0.007
	Precuneus	Parietal Lobe	BA 7	20	−24 −58 49	8.21	4.98	*p*(FWE) = 0.008
	Medial frontal gyrus	Frontal Lobe	BA 10	9	−3 50 −5	7.89	4.85	*p*(FWE) = 0.014
	Inferior frontal gyrus	Frontal Lobe	BA 46	6	−45 29 16	7.79	4.81	*p(*FWE) = 0.016
	Superior temporal gyrus	Temporal Lobe	BA 38	5	45 20 −23	7.79	4.81	*p*(FWE) = 0.016
	Hippocampus	Limbic Lobe	Hippocampus	5	−30 −16 −17	7.47	4.68	*p*(FWE) = 0.029
Main effect of Group (F contrast) negative distractors relative to no distraction	Cuneus	Occipital Lobe	BA18	247	−3 −79 22	13.88	4.63	*p*(FWE) = 0.031
	Lingual Gyrus	Occipital Lobe	BA19		−15 −61 −5	10.65	3.97	
	Posterior Cingulate	Limbic Lobe	BA30		−15 −64 4	9.34	3.67	
	Inferior Frontal Gyrus	Frontal Lobe	BA9	102	−48 5 28	12.08	4.27	*p*(FWE) = 0.010*
	Inferior Frontal Gyrus	Frontal Lobe	BA44		−54 8 19	11.08	4.07	
	Insula	Sub-Lobar	BA13		−42 11 19	7.92	3.32	

All *z* values were determined by an initial cluster-forming threshold of *p* < 0.05 family-wise error (FWE) corrected on a whole-brain voxel-wise level. Clusters detected after small volume correction (SVC) (*p* < 0.05) are indicated by an asterisk (*)

#### PPI analysis

Significant group differences were observed for amygdala FC with clusters comprising bilateral fusiform gyrus, culmen, superior/medial frontal gyrus and middle frontal gyrus, right superior/middle temporal gyrus (insular cortex) and cingulate gyrus, left inferior parietal lobule (insular cortex) and anterior insula (*p* < 0.05, FWE-cluster-corrected), right middle occipital gyrus, and left claustrum (at *p* < 0.001, uncorrected) (see Supplemental Table S1). HC showed (marginally) negative amygdala FC, while BPD groups showed positive amygdala FC with all of these regions. BPD_D differed from HC across all regions. BPD_N differed from HC regarding all regions except from middle occipital gyrus and superior temporal gyrus.

**Fig. 3:**
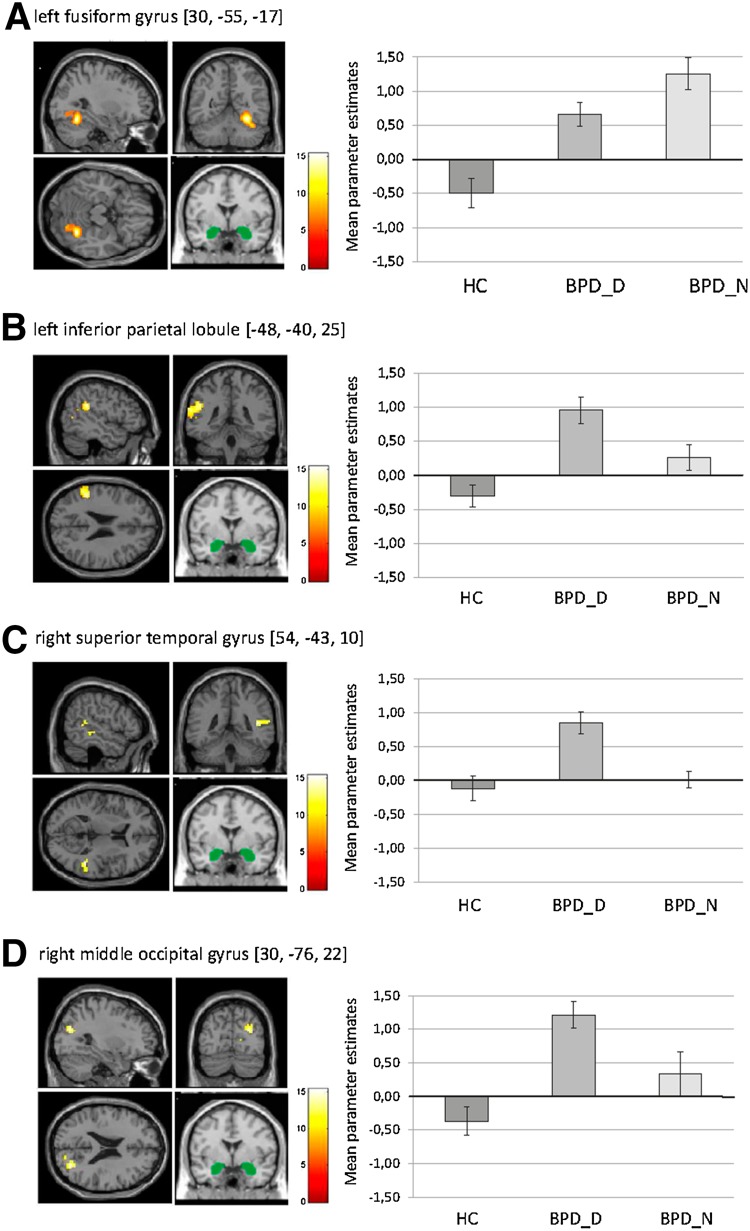
Results of the psychophysiological interaction analysis for functional connectivity (FC) of the bilateral amygdala (seed region of interest, depicted in *green*) during negative distractors versus no distraction in the context of the Emotional Working Memory Task in patients with borderline personality disorder (BPD) after dissociation induction (BPD_D) and after the neutral script (BPD_N) as well as in healthy controls (HC). The figure shows means ± standard errors of the mean of parameter estimates for bilateral amygdala FC with **a** left fusiform gyrus, **b** left inferior parietal lobule, **c** right superior temporal gyrus, and **d** right middle occipital gyrus

Compared to BPD_N, BPD_D showed reduced FC with left fusiform gyrus (*t* = 2.07, *p* = 0.048, see Fig. 3a), while showing a stronger coupling between amygdala and left inferior parietal lobule (*t* = 2.48, *p* = 0.020), right superior/middle temporal gyrus (*t* = 2.20, *p* = 0.036), and right middle occipital gyrus (*t* = 2.39, *p* = 0.024) (see Fig. 3b–d).

The MANCOVA with WM errors as covariate revealed similar results: compared to BPD_N, BPD_D showed a significantly stronger coupling between amygdala and left inferior parietal lobule (*F*_(1,26)_ = 5.96, *p* = 0.022), right superior/middle temporal gyrus (*F*_(1,26)_ = 2.54, *p* = 0.046), and right middle occipital gyrus (*F*_(1,26)_ = 4.86, *p* = 0.034), albeit group differences in amygdala FC with left fusiform gyrus were at a trend level (*F*_(1,26)_ = 2.25, *p* = 0.063)

## Discussion

The aim of our study was to investigate the impact of dissociation on brain activity and amygdala functional connectivity (FC) during emotional distraction in the context of a delay-response WM task in un-medicated patients with BPD. Using script-driven imagery, dissociation was induced in 17 BPD patients (‘BPD_D’), while 12 patients (‘BPD_N’) and 18 HC were exposed to neutral scripts. Afterwards, participants performed an Emotional Working Memory Task (EWMT) with negative vs. neutral interpersonal images versus no distractors. Main findings were:

- *Behavioral performance* Overall WM impairments (more incorrect responses and misses) in BPD_D compared to the other groups.
- Overall deactivation in the bilateral amygdala and diminished activity in the left cuneus, lingual gyrus, and posterior cingulate during emotional distraction in BPD_D compared to BPD_N; stronger left inferior frontal gyrus activity in BPD_D than in HC.
- *Amygdala FC during negative vs. no distractors* Increased amygdala connectivity with left inferior parietal lobule and right middle/superior temporal gyrus, but diminished amygdala FC with fusiform gyrus in BPD_D compared to the other groups.

The finding of impaired WM in BPD_D is consistent with previous research, pointing to detrimental effects of pathological dissociation on neuropsychological processes, such as learning, memory, attention, and interference inhibition [13, 53–55]. Since dissociation seems to influence neuropsychological functioning in BPD, dissociative symptoms should be taken into account in future experimental studies on affective–cognitive processing in BPD, even when it is not the major focus of research.

Consistent with our previous studies [27, 28], the presentation of distractors in the EWMT elicited significant activity in brain regions implicated in emotion processing, attention, WM, and interference inhibition [10, 56]. During negative vs. no distractors, the two BPD groups showed different patterns of brain activity compared to HC: BPD_N patients exhibited increased activity in amygdala and insula as well as a hyper-connectivity of the amygdala, resembling previous neuroimaging findings in BPD [6–8].

Of note, BPD patients after dissociation induction did not differ significantly from HC, while showing significantly less amygdala activity compared to BPD_N. As BPD groups were comparable regarding symptom severity, childhood trauma, PTSD comorbidity, anxiety, depressive mood, and basic working memory performance, findings point to a dampening effect of dissociation on amygdala reactivity, as proposed in current conceptualizations [15] [20].

During negative vs. no distractors, BPD_D further showed significantly lower activity in left cuneus, precuneus, and posterior cingulate—areas of the default mode network that have been implicated in self-referential processing (e.g., autobiographical memory, rumination) [57–59]. Decreased activity in these regions may suggest reduced processing of task-irrelevant—but probably self-relevant—negative social material (reminders of interpersonal violence) in patients after dissociation induction.

Consistent with previous script-driven imagery studies [21, 29] and largely in line with our hypothesis, BPD_D patients showed stronger left inferior frontal gyrus activity than HC. However, no differences in mPFC and ACC were found and increased inferior frontal gyrus activity was not specific to BPD_D (i.e., also present in BPD_N). As BPD_N did not differ significantly from HC in WM, stronger recruitment of the inferior frontal gyrus in this group may reflect compensatory efforts to prevent the occurrence of interference disinhibition on a behavioral level [60, 61].

Extending previous research, we investigated how bilateral amygdala activity was correlated to activity in other brain areas during negative vs. no distractors. Both BPD groups differed significantly from HC in amygdala FC with frontal, temporal, occipital, and parietal areas. HC showed negative amygdala connectivity with these regions, resembling findings of previous fMRI studies using the EWMT or similar tasks [28, 62, 63], while BPD patients showed positive amygdala FC with these areas. Amygdala hyper-connectivity with frontal regions, including the ACC and mPFC, was also observed in previous research and may reflect disturbed emotion processing in patients with BPD [64–69].

Importantly, we observed significant differences in amygdala connectivity between the two BPD groups, dependent on our experimental manipulation. Compared to the other groups, BPD patients exposed to the dissociation script showed diminished amygdala connectivity with left fusiform gyrus, which has been associated with encoding/processing of negative social material [70, 71]. BPD_D patients further showed a stronger coupling of the amygdala with clusters comprising right middle/superior temporal gyrus and left inferior parietal lobule. The superior temporal gyrus has previously been implicated in depersonalization and derealization [22–24] and is considered an important structure in a pathway including the amygdala and PFC, implicated in processing of language, social information, and self-perception [72]. In previous studies, higher self-reported dissociation was correlated to reduced gray matter volume [73] and increased activity in the middle/superior temporal gyrus [21] in BPD. The inferior parietal lobule has been implicated in emotion regulation and working memory—an increased information exchange of the amygdala with these areas may underlie altered emotional and self-referential processing during dissociation [74–78].

In summary, our neuroimaging findings suggest that a deactivation of the amygdala and altered interactions of this region with areas implicated in self-referential processing, cognitive control, visual perception, and sensory gating may contribute to dissociative states in BPD, while the precise mechanisms underlying stress-related dissociation remain elusive. More research is needed to clarify whether the neural patterns, observed in this study, reflect enhanced attempts to modulate states of arousal, as suggested by previous neuroimaging research in the dissociative subtype of PTSD [15] and models proposing that dissociation is a protective regulatory strategy in extremely stressful situations [17]. Dissociative responses may be an adaptive process when ‘fight or flight’ [83] is impossible [15, 17, 80], possibly stemming from an evolutionary older ‘freezing system’ [79–82]. However, the present findings provide further evidence that dissociation can become maladaptive by hindering a coherent processing of salient sensory, affective, and cognitive information in memory, which is crucial to a flexible adaptation to stressful situations [76–78]. Moreover, dissociation might not only dampen negative emotions but also positive emotions, which can have detrimental consequences for the quality of life and the maintenance of close relationships. Given these detrimental effects and previous findings of poor treatment outcome in BPD patients with pathological dissociative symptoms [18, 19, 84], our findings highlight the importance of taking dissociative symptoms into account when treating individuals with BPD.

To our knowledge, this is the first study in BPD revealing a significant impact of a dissociation induction on amygdala activity and functional connectivity during emotional distraction in the context of the EWMT. Present findings may shed a new light on stress-related dissociation in BPD, as affective–cognitive processing was studied both on a behavioral and neural level in an experimental setting which requires conscious manipulation of stressful (trauma-related) material in WM. Patient groups were matched regarding psychopathology and basic working memory and it was ensured that BPD_N patients were not dissociated. However, this led to a relatively small sample size and only female patients with a history of childhood abuse/neglect were included. We did not apply additional drug tests to rule out this possibility of false self-reports of our participants. Furthermore, it is likely that present findings may not be specific to BPD but also observable in other clinical populations with dissociative features, being a trans-diagnostic phenomenon [15, 75]. This means, more research with larger sample sizes, clinical control groups, and extended medical checks is needed to clarify whether the reported neural patterns can be replicated in other samples of BPD patients or are confounded by the afore-mentioned sample characteristics. As we used PPI, findings are restricted to our seed region and causality of interactions remains unknown [51, 52]. Tension ratings were significantly higher in BPD_D than BPD_N. Nevertheless, group differences in amygdala reactivity remained significant after including aversive tension as covariate.

All in all, our findings suggest a dampening effect of dissociation on activity in brain areas implicated in the processing of disturbing (trauma-related) information in BPD and an impairing effect on working memory, which plays a crucial role in goal-directed behavior. More research is needed to understand the impact of dissociation on other aspects of emotion regulation, cognition and identity in BPD and to gain more insight into this complex phenomenon.

## Electronic supplementary material

Below is the link to the electronic supplementary material.

**Supplemental Figure 1**: Specific types of errors (total number of errors, incorrect responses, too early responses (before probe) and misses) during the Emotional Working Memory Task in patients with borderline personality disorder (BPD) after dissociation induction (BPD_D) and after the neutral script (BPD_N) as well as in healthy controls (HC). (TIFF 90 kb)

Supplementary material 2 (DOC 89 kb)
