# Supplementary figures and images for: Reduced amygdala reactivity and impaired working memory during dissociation in borderline personality disorder
Source: Eur Arch Psychiatry Clin Neurosci. 2017 May 19;268(4):401–15. doi: 10.1007/s00406-017-0806-x (PMC5956011; doi:10.1007/s00406-017-0806-x)

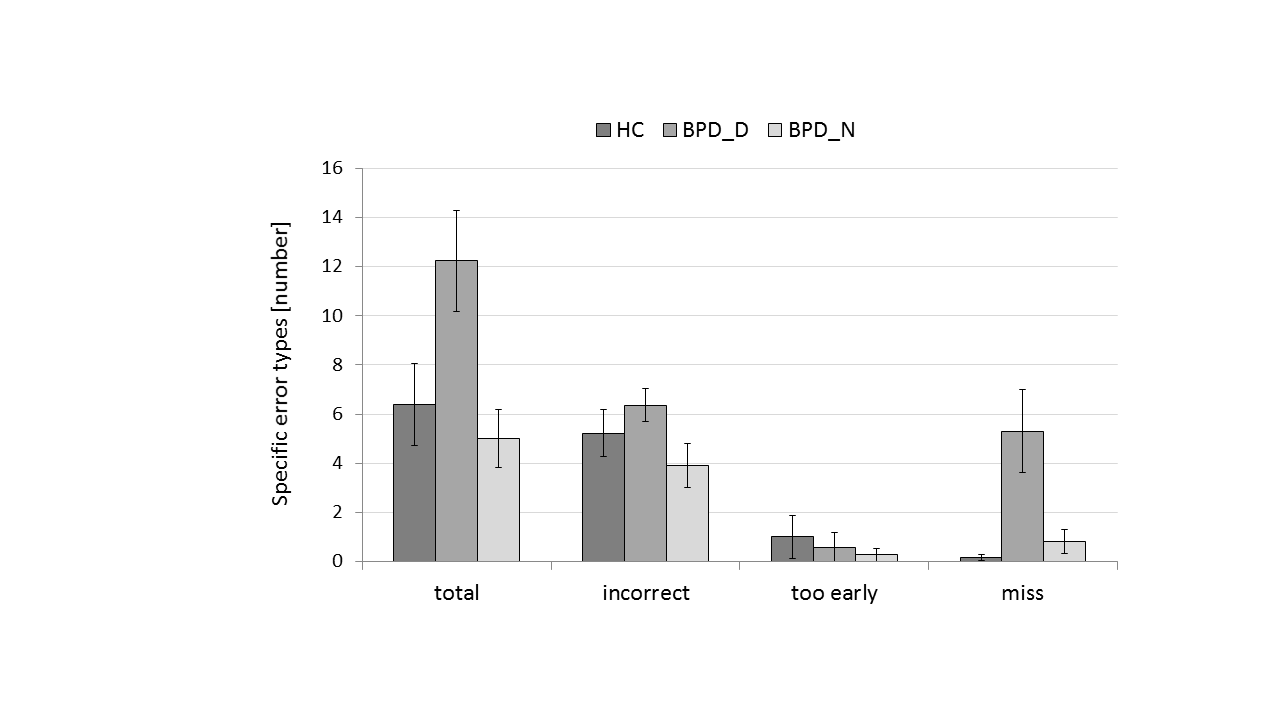

Supplement: Supplementary file 1 — Supplemental Figure 1: Specific types of errors (total number of errors, incorrect responses, too early responses (before probe) and misses) during the Emotional Working Memory Task in patients with borderline personality disorder (BPD) after dissociation induction (BPD_D) and after the neutral script (BPD_N) as well as in healthy controls (HC). (TIFF 90 kb) [file 406_2017_806_MOESM1_ESM.tif]
