# Supplementary material for: Reduced amygdala reactivity and impaired working memory during dissociation in borderline personality disorder
Source: Eur Arch Psychiatry Clin Neurosci. 2017 May 19;268(4):401–15. doi: 10.1007/s00406-017-0806-x (PMC5956011; doi:10.1007/s00406-017-0806-x)
Supplement: Supplementary file 2 — Supplementary material 2 (DOC 89 kb) [file 406_2017_806_MOESM2_ESM.doc]

**Results of the Psychophysiological Interaction (PPI) Analysis for bilateral amygdala functional connectivity during negative versus no** distractors

| Label of brain region (aal) | Lobe | Brodman area (BA) | Cluster size | Peak voxel coordinates  (MNI: X, Y, Z) | F value | Z value | P value |
| --- | --- | --- | --- | --- | --- | --- | --- |
| Fusiform Gyrus  Fusiform Gyrus  Culmen | Occipital Lobe  Posterior Lobe  Anterior Lobe | N.A.  N.A.  N.A. | 1118 | 30 -55 -17  -27 -55 -14  39 -52 -29 | 21.91  18.27  16.99 | 5.03  4.65  4.50 | p(FWE) <0.05 |
| Superior Frontal Gyrus,  Medial Frontal Gyrus,  Supplemental Motor Area | Frontal Lobe  Frontal Lobe  Frontal Lobe | BA 6  BA 6  BA 6 | 457 | 12 2 70  3 -7 58  -3 5 61 | 21.36  17.09  16.46 | 4.97  4.51  4.43 | p(FWE) <0.05 |
| Middle Frontal Gyrus  Middle Frontal Gyrus | Frontal Lobe  Frontal Lobe | BA 6  BA 6 | 127 | 42 -4 55  27 -7 64 | 20.45  9.15 | 4.88  3.30 | p(FWE) <0.05 |
| Inferior Parietal Lobule/  Supramarginal Gyrus  Temporal Gyrus  (Insular cortex) | Parietal Lobe  Parietal Lobe  Temporal Lobe | BA 40  BA 13  BA 21 | 163 | -57 -46 22  -48 -40 25  -63 -52 7 | 14.95  13.45  9.85 | 4.24  4.03  3.44 | p(FWE) <0.05 |
| Precentral Gyrus  Middle Frontal Gyrus | Frontal Lobe  Frontal Lobe | BA 6  BA 6 | 76 | -42 -4 43  -36 -1 61 | 14.24  11.05 | 4.14  3.65 | *p*(uncor) <0.001 |
| Insula  Superior Temporal Gyrus  Superior Temporal Gyrus | Sub-lobar  Temporal Lobe  Temporal Lobe | BA 13  BA 38  BA 38 | 119 | 39 14 -2  57 5 -8  51 17 -8 | 13.92  13.89  13.53 | 4.10  4.10  4.04 | p(FWE) <0.05 |
| Insula | Sub-lobar | N.A. | 16 | -33 11 -2 | 13.12 | 3.98 | p(FWE) <0.05* |
| Claustrum  Claustrum | Sub-lobar  Sub-lobar | N.A.  N.A. | 46 | -30 11 -5  -27 26 -2 | 13.72  8.62 | 4.07  3.20 | *p*(uncor) <0.001 |
| Middle Occipital Gyrus | Occipital Lobe | BA 31 | 50 | 30 -76 22 | 12.26 | 3.85 | *p*(uncor) <0.001 |
| Superior Temporal Gyrus  Superior Temporal Gyrus | Temporal Lobe  Temporal Lobe | BA 41  BA 22 | 34 | 45 -43 10  54 -43 10 | 11.91  9.34 | 3.80  3.34 | *p*(uncor) <0.001 |
| Superior Frontal Gyrus | Frontal Lobe | BA 8 | 10 | -3 32 58 | 10.83 | 3.62 | p(FWE) <0.05* |
| Middle Temporal Gyrus | Temporal Lobe | N.A. | 11 | 51 -34 -5 | 10.30 | 3.52 | *p*(uncor) <0.001 |
| Cingulate Gyrus | Limbic Lobe | BA 32 | 34 | 6 11 43 | 13.27 | 4.01 | p(FWE) <0.05* |
| Superior Frontal Gyrus | Frontal Lobe | BA 8 | 10 | -3 32 58 | 10.83 | 3.62 | p(FWE) <0.05* |

Note: Z -values were determined by an initial cluster-forming threshold of p<0.001 uncorrected (uncor) on a whole-brain voxel-wise level. FWE = Family Wise Error corrected at a cluster level. Clusters detected after small volume correction (SVC) (p<0.05) are indicated by an asterisk (*).
